# Building gene expression profile classifiers with a simple and efficient rejection option in R

**DOI:** 10.1186/1471-2105-12-S13-S3

**Published:** 2011-11-30

**Authors:** Alfredo Benso, Stefano Di Carlo, Gianfranco Politano, Alessandro Savino, Hafeez Hafeezurrehman

**Affiliations:** 1 Control and Computer Engineering Department, Politecnico di Torino, Corso Duca degli Abruzzi 24,10129, Torino, Italy

## Abstract

**Background:**

The collection of gene expression profiles from DNA microarrays and their analysis with pattern recognition algorithms is a powerful technology applied to several biological problems. Common pattern recognition systems classify samples assigning them to a set of known classes. However, in a clinical diagnostics setup, novel and unknown classes (new pathologies) may appear and one must be able to reject those samples that do not fit the trained model. The problem of implementing a rejection option in a multi-class classifier has not been widely addressed in the statistical literature. Gene expression profiles represent a critical case study since they suffer from the curse of dimensionality problem that negatively reflects on the reliability of both traditional rejection models and also more recent approaches such as one-class classifiers.

**Results:**

This paper presents a set of empirical decision rules that can be used to implement a rejection option in a set of multi-class classifiers widely used for the analysis of gene expression profiles. In particular, we focus on the classifiers implemented in the R Language and Environment for Statistical Computing (R for short in the remaining of this paper). The main contribution of the proposed rules is their simplicity, which enables an easy integration with available data analysis environments. Since in the definition of a rejection model tuning of the involved parameters is often a complex and delicate task, in this paper we exploit an evolutionary strategy to automate this process. This allows the final user to maximize the rejection accuracy with minimum manual intervention.

**Conclusions:**

This paper shows how the use of simple decision rules can be used to help the use of complex machine learning algorithms in real experimental setups. The proposed approach is almost completely automated and therefore a good candidate for being integrated in data analysis flows in labs where the machine learning expertise required to tune traditional classifiers might not be available.

## Background

Microarrays are one of the latest breakthroughs in experimental molecular biology. They allow to simultaneously monitoring the expression level of tens of thousands of genes. Arrays coupled with pattern recognition methods have been applied to studies in gene expression, genome mapping, transcription factor activity, toxicity, pathogen identification, and many other applications [1-10]

However, although in standard classification problems one has to classify a sample and assign it to one of a set of known classes, in a clinical diagnostics setup in which some classes (phenotypes) may be known but novel unknown classes (new phenotypes) may appear as well, one must be able to reject those samples that do not fit the trained model.

In this paper, we present a set of empirical decision rules designed to implement a rejection option in a set of multi-class classifiers widely used for the analysis of gene expression profiles. In particular, we will focus on the R Language and Environment for Statistical Computing (R for short in the remaining of this paper) [11].

The problem of implementing a rejection option in a multi-class classifier has not been widely addressed in the statistical literature with the exception of a few publications [12-15]. Chow [12] put forth the decision theoretic framework to rejection in pattern recognition. The overall idea is to estimate the class conditional probabilities for a sample and to reject it if the maximum probability is below a given threshold. This simple rejection rule is optimal when the class conditional probabilities can be estimated without errors, which is in contrast with several real setups [16]. Gene expression profiles suffer from the curse of dimensionality problem [17] that negatively reflects on the reliability of probability estimators. The number of available classes and the correct setup of the threshold are additional constraints that limit the reliability of this approach. An attempt to setup per-class thresholds has been proposed by Fumera et al. [18] to mitigate errors in probability estimation. However, the computational effort and the complexity of tuning the resulting classification system increases, limiting a widespread application in laboratory setups. Recently, one-class classifiers gained attention in the implementation of rejection systems in gene expression profiles [19-22]. These algorithms base the prediction model on the concept of distance among samples rather than on the estimation of class conditional probabilities. They therefore overcome the limited reliability of available class probability estimators. However, increased number of classes, high dimensionality feature spaces such as the one of microarray datasets, noisy features, and quite often not enough samples, still limit their accuracy.

In this paper we will build a set of rejection rules able to work with the very simple and often unreliable class probability estimators provided with the multi-class classifiers implemented in R (see the Methods section for further details). The main contribution of the proposed rules is their simplicity. It makes possible an easy integration with available data analysis environments while maintaining, at the same time, good classification performance. Since in the definition of a rejection model tuning of the involved parameters is often a complex and delicate task, in this paper we exploit an evolutionary strategy to automate this process. This allows the final user to maximize the rejection accuracy with minimum manual intervention.

A complete experimental setup is presented to validate the proposed model on a challenging data-set of blood diseases. A set of three multi-class classifiers widely adopted in the analysis of gene expression profiles which are also available in R has been considered. Results are compared to those obtained building rejection options based on one-class classifiers [23]. Results show that the proposed decision rules can be efficiently used as a powerful rejection method, outperforming most of the considered one-class classifiers.

## Results and discussion

### Experimental setup

The results of this paper have been validated on a dataset of gene expression profiles from complementary DNA (cDNA) microarrays related to very similar phenotypes. Only a reduced subset of genes allows for discrimination (Table 1). This peculiarity increases the complexity of the classification allowing us to better validate the proposed method. It is worth mentioning here that, in all experiments, the training-set does not include any sample from the test-set. This is a given requirement to avoid overoptimistic results and therefore to honestly evaluate the classifiers performances.

**Table 1:** Structure of the considered data-set

Blood data-set	
**Phenotype acronym**	**#Samples**

DLBCL	61
CLLww	31
CLL	21
ALL	27
CBF-AML	21
FL	24
AML2	11
Total	196

The table reports the acronym of each phenotype and the related number of samples.

The data-set includes a total of 7 phenotypes. Samples have been downloaded from the cDNA Stanford Microarray database [24]. All genes without a valid UnigeneID have been discarded. The expression level of each gene is measured as the log-ratio between the Cy5 and the Cy3 channel of the array: .

Four sets of samples have been downloaded from a large set of experiments aiming at performing Lym-phoma Classification [25,26]:

• Diffuse Large B-Cell Lymphoma (DLBCL): a non-Hodgkin lymphoma disease,

• B-Cell Chronic Lymphocytic Leukemia Wait&Watch (CLLww),

• B-Cell Chronic Lymphocytic Leukemia (CLL), and

• Follicular Lymphoma (FL): independent lymphonode samples on LymphoChip microarrays [27].

The remaining three phenotypes in the data-set are:

• Acute Lymphoblastic Leukemia (ALL),

• Core Binding Factor Acute Myeloid Leukemia (CBF-AML): subgroups characterized by shorter overall survival [28],

• Acute Myeloid Leukemia 2 data-set (AML2): peripheral-blood samples or bone marrow samples of intermediate-risk AML with a normal karyotype.

Three multi-class classifiers often used in gene expression profile analysis have been considered in this study: k–Nearest Neighbors (k-NN), feed-forward Neural NETwork with a single hidden layer (N-NET), and Random Forests (RF). All algorithms are available in R. A detailed description of how data have been processed and how the prediction models for the different classifiers have been trained is available in the Methods section.

### Class probability estimates analysis and decision rules

The process of detecting samples to reject in a multi-class classification system can be modeled as a binary classification test discriminating between samples that belong to one of the known classes (*target samples*) and samples that do not belong to any of them (*reject samples*). The outcome of the test is measured in terms of:

• *true positives* (TP): target samples correctly accepted,

• *true negatives* (TN): reject samples correctly rejected,

• *false positives* (FP): reject samples erroneously accepted, and

• *false negatives* (FN): target samples erroneously rejected.

The number of TP, TN, FP, and FN adds up to 100% of the data-set. The accuracy in which target samples are assigned to the corresponding class is out of the scope of this work and depends on the accuracy of the selected multi-class classifier.

The multi-class classifiers considered in this paper (RF, N-NET and k-NN) do not natively implement a rejection option. Discarding reject samples by setting a single threshold on the class probability estimates is inaccurate since class probability estimates show small differences between target and reject samples (refer to the Methods section for specific details on how class probability estimates have been computed). However, this information can still be used for discrimination if coupled with well tuned decision rules.

**Figure 1:**
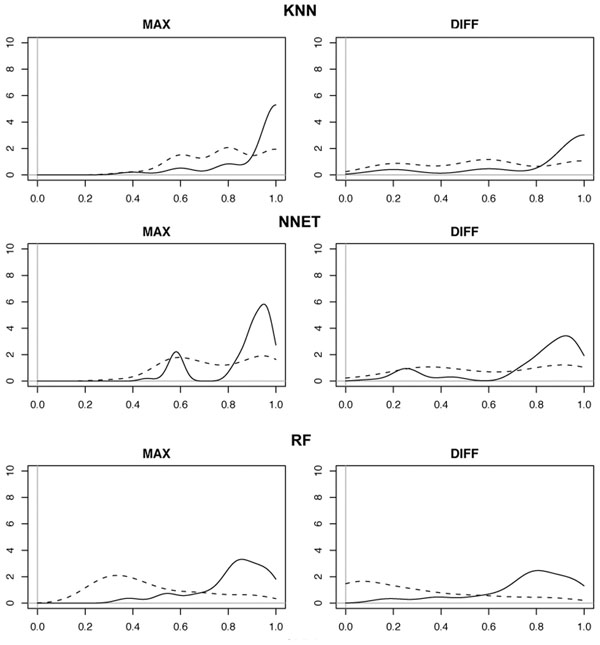
**Probability density plots.** Probability density plots to show how the value of class probability estimates of target and reject samples distribute over a set of experiments. In the MAX plot the considered random variable is the highest class probability estimate of each classified sample, split into target samples (solid line) and reject samples (dashed line). In the DIFF plot the considered random variable is the difference between the two highest probability estimates of each sample, considering target samples (solid line) and reject samples (dashed line).

In order to perform a preliminary qualitative analysis of how class probability estimates change between target and reject samples, we performed a set of multi-class classification experiments generating different splits of the considered data-set (in terms of targets/reject samples and test/training data). For each split, the multi-class classifiers have been trained on a subset of the considered phenotypes, using the remaining data as a set of samples to reject. Figure 1 reports, for each classifier, two density plots that show how the value of class probability estimates of target and reject samples distribute in the performed experiments. In the MAX plot the considered random variable is the highest class probability estimate of each classified sample, split into target samples (solid line) and reject samples (dashed line); in the DIFF plot the considered random variable is the difference between the two highest probability estimates of each sample, again considering target and reject samples. The density functions have been estimated from the experimental data by performing a Gaussian kernel density estimation using the density() command of R.

Although the plots of Figure 1 may seem to suggest a strong overlap between the distributions of target samples (solid lines) and reject samples (dashed lines), a certain amount of separation is still visible. This is particularly evident in the case of RF, that shows a quite visible distinction both in the MAX and in the DIFF plots. In particular, in the DIFF plot of RF, target samples (solid line) have a max around 0.8, far from the max of reject samples (dashed line) that falls around 0.1. This means that, for a target sample, the difference between the two top rated classes is very high (around 0.8 in most of the cases). Instead, reject samples show a very low difference between probability estimates of the two top ranked classes, revealing the inability of the classifier to clearly select a target class. k-NN and N-NET show smaller separation; however, experimental results will show that a partial discrimination is still possible.

**Figure 2:**
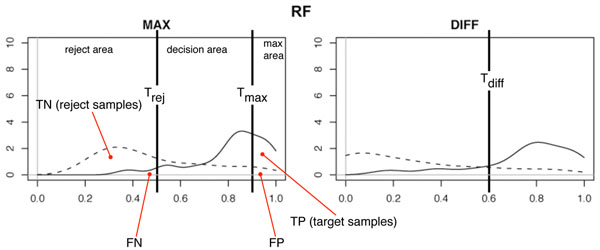
**Definition of the rejection thresholds in the case of RF**. The MAX plot is split into three distinct zones: (i) max area, (ii) decision area, and (iii) reject area.

From this preliminary analysis, it seems reasonable that, for the three considered classifiers, the class probability estimates provided by R could potentially be used for detecting reject samples. The idea exploited in this paper to design a set of decision rules for detecting reject samples is to split the MAX plot into three distinct areas: (i) max area, (ii) decision area, and (iii) reject area, delimited by two rejection thresholds *T_max_* and *T_rej_* (*T_max_* >*T_rej_*), as shown in Figure 2 for RF.

**Figure 3:**
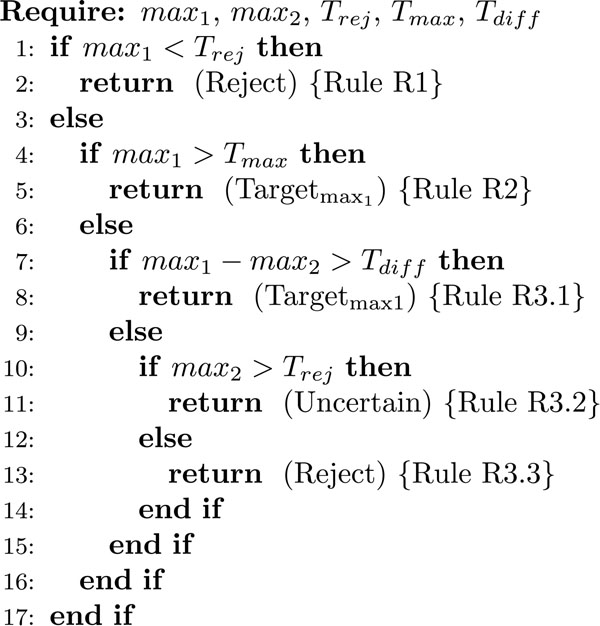
**Decision Rule Pseudo-code**. Pseudo-code describing the decision rules able to discriminate between target and reject samples based on the class probability estimates of the sample and on the computed rejection thresholds.

Figure 3 describes the overall decision process applied to each sample that must be classified. *max*_1_ and *max*_2_ denote the two highest class probability estimates for the considered sample.

If the highest class probability estimate (*max*_1_) is lower than *T_rej_*, then the sample falls in the reject area and is rejected to maximize the number of TN (Rule R1 Figure 3, rows 1-2). Similarly, if *max*_1_ is higher than *T_max_*, the sample falls in the max area and can be accepted to maximize the number of TP. The class with probability estimate equal to *max*_1_ is predicted (Rule R2-Figure 3, rows 4-5). The first part of this decision process is very similar to the single threshold method proposed in [12].

Whenever neither R1 or R2 are satisfied, i.e., *max*_1_ falls between *T_rej_* and *T_max_* (decision area) there are two possible conditions based on the analysis of the difference between *max*_1_ and *max*_2_ (DIFF plot of Figure 2):

1. if *max_1_* – *max*_2_ >*T_diff_*, the sample can be accepted and the class with probability estimate equal to *max*_1_ is predicted (Rule R3.1-Alg. 3, rows 7-8). *T_diff_* is the minimum difference between the probability estimates of the two top ranked classes that allows us to use *max*_1_ to perform a reliable classification;

2. if *max_1_* – *max*_2_ <*T_diff_* , the value of *max*_2_ is considered. Two cases are possible:

• if *max*_2_ is higher than *T_rej_*, i.e., both *max*_1_ and *max*_2_ fall in the decision area, the prediction is considered *uncertain* (Rule R3.2-Alg. 3, rows 10-11). In this case the classifier does not produce any classification result. This rule prevents from providing a result when the distinction between two classes is not sufficient to correctly discriminate. In alternative, multiple classification results can be provided to alert the user that the confidence in the prediction is low;

• if *max*_2_ is lower than *T_rej_*, the sample is rejected (Rule R3.3-Alg. 3, row 13). This rule mitigates the noise in those samples that fall at the border of the reject area.

The three rejection thresholds (*T_max_*, *T_rej_*, and *T_diff_*) can be empirically chosen analyzing the density plots of Figure 2:

• if the MAX plot shows a clear separation between target and reject samples, *T_max_* can be placed in such a way to maximize the number of TP immediately detected by rule R2;

• similarly to *T_max_*, *T_rej_* can be placed to maximize the number of TN detected by rule R1;

• the definition of *T_diff_* is performed looking at the DIFF plot. A good heuristic is to consider the point where the two curves intersect.

Manually setting the three thresholds is very complex and may easily lead to a high error rate. When the plots do not show a clear separation between target and reject samples, the choice of the thresholds involves a trade-off between increasing the sensitivity, and lowering the specificity of the classifier. This is a complex optimization task.

All thresholds must be setup only considering information extracted from the considered training data. To tackle the complexity of this process, and to allow the automatic tuning of all rejection parameters, a threshold setup algorithm based on a Covariance Matrix Adaptation Evolutionary Strategy (CMA-ES) has been developed. The full description of this algorithm is available in the Methods section.

### Architecture of the multi-class classifier with rejection option

**Figure 4:**
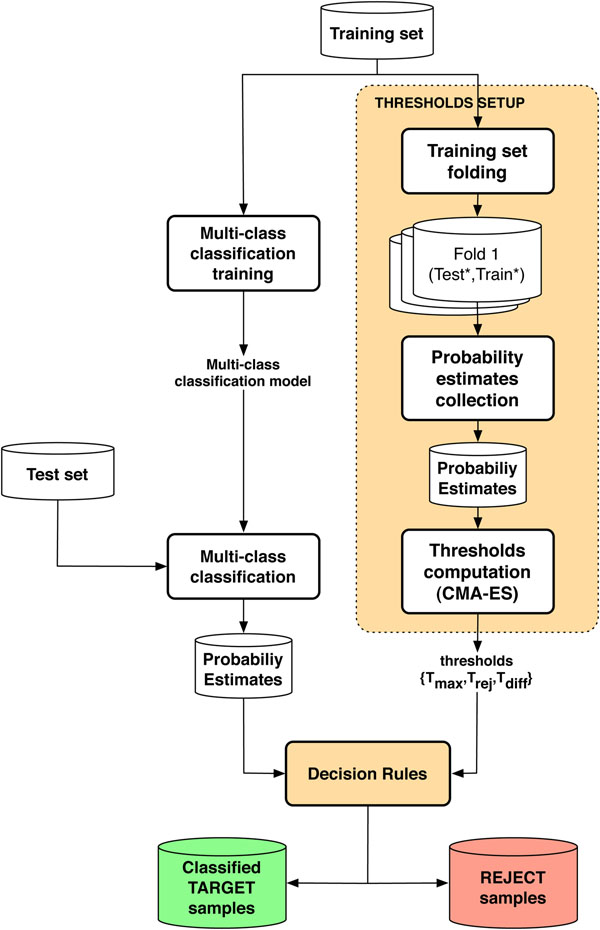
**Computational flow**. Computational flow to setup a multi-class classification system with a rejection option based on the proposed decision rules.

The proposed decision rules can be easily integrated within the multi-class classification flow provided by R or other similar computational environments. Figure 4 shows the computational flow of the resulting system. As usual when working with classification algorithms, a training set containing known samples is used to train the prediction model then used to classify a set of test (unknown) samples.

Compared to a traditional multi-class classification flow, the proposed system includes two additional modules required to: 1) setup the rejection thresholds, and 2) apply the decision rules.

**Figure 5:**
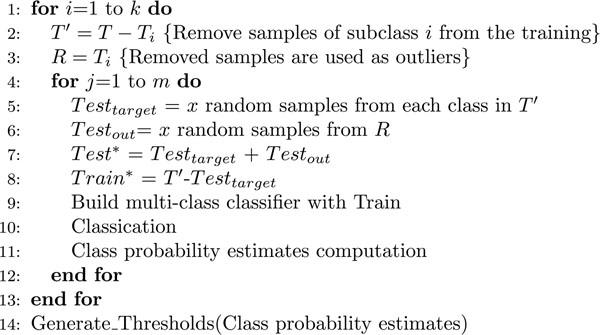
Pseudo-code description of the threshold setup process.

Setting up the rejection thresholds requires collecting a statistically significant set of class probability estimates for both target and reject samples on which to compute the density plots of Figure 2. At this stage, in which the model is trained and real reject samples are not available, this information can only be collected starting from samples in the training set by setting up several cross-validation experiments on different folds of these training data. Figure 5 outlines the way this module operates. Let us denote with *T* the full training set and with *T_i_* a portion of it including only those samples related to a specific phenotype. If *k* classes are included in *T*, *k* subsets of experiments are generated by iteratively excluding one of the classes *T_i_* from *T* to form a new target class *T*′. The removed samples are used as a set of reject samples denoted with *R* (Figure 5, rows 1-3).

For each subset (Figure 5, row 4), *m* folds are generated by removing *x* samples from each subclass contained in *T′*, and *x* samples from *R*. Each fold will therefore generate a test-set (Test*) of *x* · (*k* – 1) target samples , and *x* reject samples. To avoid overoptimistic results, target samples of the test-set are removed from *T*′ forming a new training set Train* (Figure. 5, rows 5-8). Each fold is then used for an independent multi-class classification experiment to obtain the class probability estimates of each test sample in Test*. After running all *k* · *m* experiments, the CMA-ES analyzes the collected probability estimates and provides a set of optimal thresholds (refer to the Methods section for a complete description of this step).

### Validation and discussion

The proposed rejection rules have been tested on different groups of experiments based on different configurations of the considered data-set in terms of target and reject samples. The rejection accuracy has been compared with the one of a set of selected one-class classifiers. Five one-class classification algorithms have been considered in this comparison:

• Parzen one-class classifier (Parzen-OC),

• k-NN one-class classifier (k-NN-OC),

• K-Means one-class classifier (k-Means-OC),

• Principal Component Analysis (PCA) one-class classifier (PCA-OC), and

• SVDD, a SVM based one-class classifier (SVM-OC).

All one-class classifiers have been implemented and optimized using Matlab’s DD_tools [29], a standard implementation used in several publications on microarray analysis [19-22].

Two methods of using one-class classifiers have been considered. Let us suppose to have a target class including *k* different subclasses (phenotypes). The one-class classification problem can be solved by training either *k* different one-class classifiers (one for each subclass) with samples rejected if rejected by all classifiers, or a single one-class classifier trained with samples of all *k* subclasses. The first approach will be referred to as Multi One-Class with Voting (MOCV) while the second approach will be referred to as Single One-Class with Multiple Targets (SOCMT).

Four groups of experiments each denoted as *G_k_* (*k* ∈ [3, 6]) have been generated. In the group *G_k_* the target class includes *k* out of the 7 available phenotypes. Samples in the remaining 7 – *k* classes must be rejected. For each group different random combinations of the *k* classes included in the target profile are considered and for each combination several random splits of data into test- and training-set are generated for a total amount of 40 experiments for each group. For all experiments, the test-set includes a balanced number of target and reject samples.

For each experiment data are classified with MOCV, SOCMT, and the three considered multi-class classifiers paired with the decision rules presented in this paper. Rejection thresholds have been automatically computed according to the process described in Figure 5.

Table 2 summarizes the results of the classification. It provides for each classifier (rows), and for each group of experiments (column groups), the average sensitivity (Sens) and specificity (Spec) with the corresponding Confidence Intervals (CI) computed with 95% Level of Confidence (LOC). RF coupled with the decision rules is the classifier that globally better performs with respect to any other available option (its performance is highlighted in bold in Table 2). The other two multi-class classifiers (N-NET and k-NN) are also comparable or in some cases better than one-class classifiers. This result can be better appreciated looking at Table 3 reporting the average accuracy improvement of the proposed approach over the two possible configurations of one-class classifiers. Accuracy is computed as the percentage of samples correctly classified (*TP* + *TN*) over the total amount of classified samples. Averages are computed over the 40 experiments of the corresponding group. Looking at the performance of RF (highlighted in bold) one can notice a significant improvement of the accuracy in all experiments compared to one-class classifiers. The table also highlights how the other two multi-class classifiers have performances comparable to one-class classifiers in most of the experiments.

**Table 2:** Classification performances

	*G*_3_				*G*_4_				*G*_5_				*G*_6_			
**Classifier**	**Sens**	**CI**	**Spec**	**CI**	**Sens**	**CI**	**Spec**	**CI**	**Sens**	**CI**	**Spec**	**CI**	**Sens**	**CI**	**Spec**	**CI**

K-NN + rule	0.70	[0.61-0.79]	0.42	[0.31-0.53]	0.70	[0.65-0.75]	0.45	[0.36-0.55]	0.59	[0.49-0.69]	0.42	[0.35-0.49]	0.55	[0.49-0.62]	0.59	[0.48-0.70]
**RF + rule**	**0.61**	**[0.53-0.69]**	**0.76**	**[0.69-0.83]**	**0.75**	**[0.71-0.79]**	**0.68**	**[0.60-0.77]**	**0.69**	**[0.66-0.73]**	**0.78**	**[0.70-0.86]**	**0.77**	**[0.70-0.84]**	**0.63**	**[0.53-0.73]**
N-NET+rule	0.38	[0.23-0.53]	0.68	[0.53-0.83]	0.80	[0.70-0.90]	0.50	[0.43-0.57]	0.57	[0.51-0.63]	0.71	[0.59-0.84]	0.81	[0.75-0.87]	0.34	[0.25-0.44]
K-NN-OC*_SOCMT_*	0.74	[0.67-0.82]	0.20	[0.17-0.23]	0.90	[0.87-0.93]	0.29	[0.23-0.35]	0.91	[0.89-0.92]	0.23	[0.21-0.25]	0.86	[0.81-0.90]	0.16	[0.10-0.21]
KMeans-OG*_SOCM_*	0.47	[0.41-0.53]	0.66	[0.60-0.73]	0.54	[0.50-0.57]	0.69	[0.62-0.76]	0.65	[0.61-0.70]	0.67	[0.63-0.71]	0.61	[0.53-0.69]	0.50	[0.38-0.62]
PCA-OC*_SOCMT_*	0.40	[0.35-0.45]	0.79	[0.77-0.81]	0.36	[0.31-0.42]	0.77	[0.69-0.84]	0.29	[0.25-0.34]	0.76	[0.69-0.82]	0.29	[0.23-0.35]	0.79	[0.72-0.86]
Parzen-OC*_SOCMT_*	0.44	[0.39-0.50]	0.70	[0.64-0.76]	0.49	[0.46-0.52]	0.73	[0.67-0.79]	0.51	[0.47-0.54]	0.77	[0.70-0.83]	0.49	[0.45-0.52]	0.79	[0.70-0.87]
SVM-OC*_SOCMT_*	0.20	[0.16-0.24]	0.72	[0.68-0.77]	0.22	[0.20-0.25]	0.76	[0.70-0.81]	0.27	[0.24-0.30]	0.77	[0.70-0.83]	0.28	[0.25-0.31]	0.80	[0.72-0.88]
s K-NN-OC*_MOCV_*	0.99	[0.98-1.00]	0.01	[0.00-0.02]	1.00	[1.00-1.00]	0.00	[0.00-0.00]	1.00	[1.00-1.00]	0.00	[0.00-0.00]	1.00	[1.00-1.00]	0.00	[0.00-0.00]
KMeans-OC*_MOCV_*	0.36	[0.29-0.43]	0.71	[0.63-0.80]	0.48	[0.43-0.54]	0.78	[0.72-0.83]	0.59	[0.57-0.61]	0.69	[0.64-0.73]	0.46	[0.44-0.49]	0.68	[0.60-0.77]
PCA-OC*_MOCV_*	0.42	[0.36-0.48]	0.71	[0.65-0.77]	0.50	[0.48-0.52]	0.78	[0.72-0.83]	0.49	[0.46-0.53]	0.77	[0.70-0.83]	0.43	[0.40-0.45]	0.70	[0.60-0.80]
Parzen-OC*_MOCV_*	0.36	[0.31-0.40]	0.70	[0.64-0.76]	0.34	[0.28-0.41]	0.78	[0.72-0.83]	0.32	[0.28-0.36]	0.77	[0.70-0.83]	0.22	[0.20-0.24]	0.70	[0.60-0.80]
SVM-OC*_MOCV_*	0.19	[0.15-0.23]	0.76	[0.74-0.79]	0.25	[0.22-0.28]	0.79	[0.73-0.85]	0.27	[0.24-0.30]	0.78	[0.72-0.84]	0.22	[0.20-0.24]	0.72	[0.63-0.81]

For each group of experiments results are provided in terms of average sensitivity (Sens) and specificity (Spec) with the related Confidence Intervals (CI). Confidence intervals are computed with 95% LOC.

**Table 3:** Average improvements

		SOCMT					MOCV				
		K-NN-OC	KMeans-OC	PCA-OC	Parzen-OC	SVM-OC	K-NN-OC	KMeans-OC	PCA-OC	Parzen-OC	SVM-OC

G_3	K-NN+rule	+0.08	+0.01	-0.01	+ 0.01	+ 0.12	+ 0.04	+ 0.05	+0.01	+0.05	+0.11
	**RF+rule**	**+0.19**	**+0.12**	**+0.10**	**+0.12**	**+0.23**	**+0.15**	**+0.16**	**+0.12**	**+0.16**	**+0.22**
	N-NET+rule	+0.03	-0.04	-0.06	-0.04	+ 0.07	-0.01	+ 0.00	-0.04	+0.00	+0.06

G_4	K-NN+rule	-0.01	-0.05	-0.01	-0.05	+ 0.06	+ 0.10	-0.07	-0.08	+0.00	+0.03
	**RF+rule**	**+0.13**	**+0.09**	**+0.13**	**+0.09**	**+0.20**	**+0.24**	**+0.07**	**+0.06**	**+0.14**	**+0.17**
	N-NET+rule	+0.06	+0.02	+ 0.06	-0.04	0.13	+ 0.17	+ 0.00	-0.01	+0.00	0.1

G_5	K-NN+rule	-0.04	-0.16	-0.05	-0.15	-0.04	+ 0.04	-0.14	-0.14	-0.06	-0.05
	**RF+rule**	**+ 0.2**	**+0.08**	**+0.19**	**+0.09**	**+0.20**	**+0.28**	**+0.1**	**+ 0.1**	**+0.18**	**+0.19**
	N-NET+rule	+0.11	-0.01	+ 0.10	+ 0.00	+ 0.11	+ 0.19	+ 0.01	+0.01	+0.09	+0.10

G_6	K-NN+rule	+0.06	+0.01	+ 0.03	-0.07	+ 0.03	+ 0.07	+ 0.00	+0.01	+0.11	+0.10
	**RF+rule**	**+0.19**	**+0.14**	**+0.16**	+ 0.06	+ 0.16	**+0.20**	**+0.13**	**+0.14**	+0.24	+0.23
	N-NET+rule	+0.07	+0.02	+ 0.04	+ 0.01	+ 0.04	+ 0.08	+ 0.01	+0.02	+0.19	+0.11

Results are provided in terms of improvement of the accuracy of multi-class classifiers with decision rules versus one-class classifiers.

**Figure 6:**
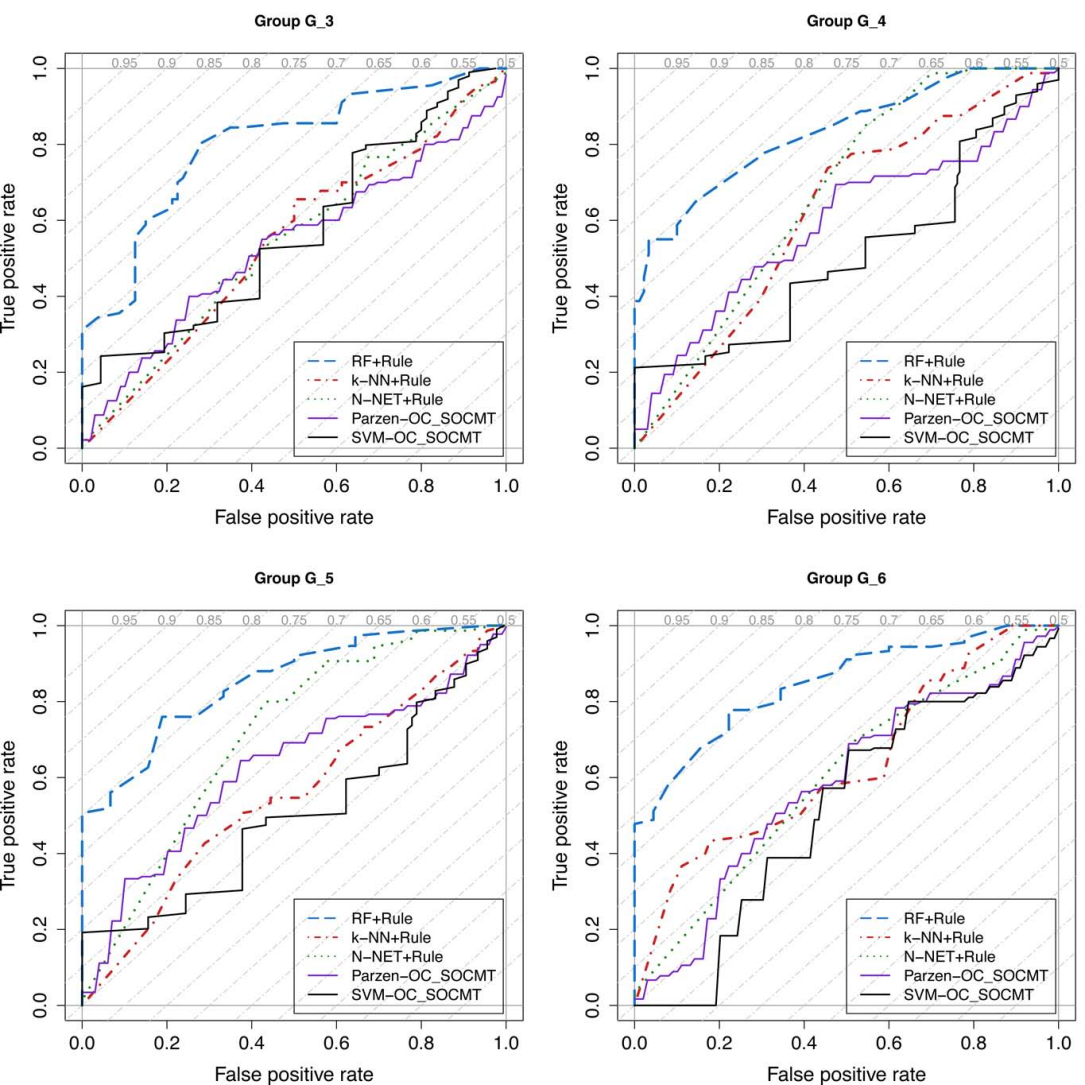
**ROC curves.** ROC curves comparing the best one-class classifiers with the multi-class classifiers coupled with the decision rules.

A final confirmation of the improvement introduced by the presented approach can be appreciated in the Receiver Operating Characteristic (ROC) curves of Figure 6. For each group of experiments the related ROC curve compares the average performance of the three multi-class classifiers coupled with the decision rules and the two best one-class classifiers (Parzen-OC and SVM-OC). In the case of multi-class classifiers the ROC curve is plotted by changing the value of the three rejection thresholds in order to explore as much as possible the space of possible solutions, while, in the case of one-class classifiers, it is obtained by changing the considered rejection rate. In all experiments RF improves the accuracy of the one-class classifiers while k-NN and N-NET provide an accuracy that is comparable to those of one-class classifiers. This result is obtained using a very simple computational model compared to the one required to setup a one-class classification model.

**Figure 7:**
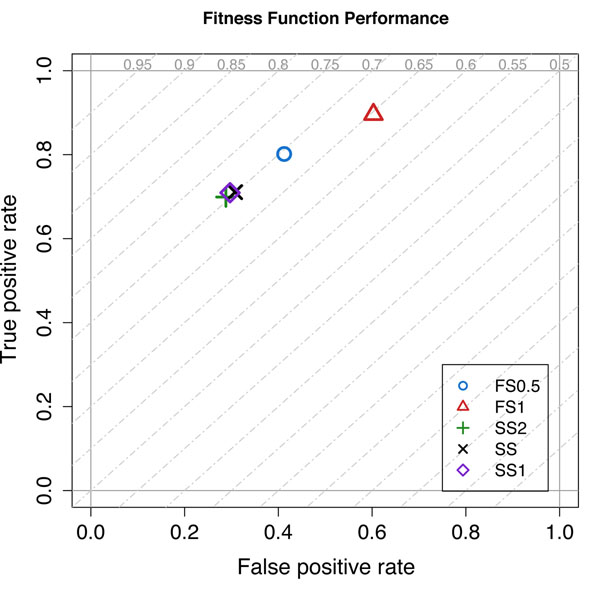
**CMA-ES Objective functions performances for RF**. ROC graphs comparing the accuracy of RF while computing the rejection thresholds with different objective functions.

All proposed results have been obtained by computing the rejection thresholds using the CMA-ES with the SS1 objective function (see the Methods section). The diagram of Figure 7 shows the average accuracy obtained during the cross-validation experiment used to setup the rejection thresholds. Proposed results are for RF coupled with the rejection rules considering the different CMA-ES objective functions. The dashed diagonal lines represent iso-accuracy lines, with accuracy that decreases from the top-left corner to the bottom-right corner. The graph shows that the three functions SS, SS1, and SS2 provide the better accuracy with SS1 providing the best results.

The value of the objective function associated with the thresholds computed by the CMA-ES can be used as an indicator of the reliability of the trained model. Whenever an objective function is equal to 0, it means perfect discrimination among target samples and reject samples. Values greater than 0 indicate reduced accuracy. This is confirmed by the results of Table 4. It reports for each classifier and for each group of experiments the average accuracy and the average value of the SS1 objective function associated with the computed thresholds. The numbers clearly show how RF, that is the one with better accuracy, has a lower value of the objective function compared k-NN and N-NET, thus suggesting a more reliable model.

**Table 4:** Reliability of the trained model

	*G*_3_		*G*_4_		*G*_5_		*G*_6_	
	acc	*f*	acc	*f*	acc	*f*	acc	*f*

RF+Rule	0.68	0.21	0.72	0.23	0.72	0.29	0.70	0.24
N-NET+Rule	0.54	0.40	0.65	0.40	0.65	0.40	0.58	0.40
k-NN+Rule	0.56	0.40	0.58	0.37	0.51	0.38	0.57	0.41

The value of the objective function is used as an indicator of the reliability of the trained model.

## Conclusions

Life sciences are undergoing a true revolution as a result of the emergence and growing impact of a series of new disciplines/tools including genomics, transcriptomics, proteomics and metabolomics. These new disciplines are devoted to the examination of the entire systems of genes, transcripts, proteins and metabolites present in a given cell or tissue type. New technologies allow now to collect huge amounts of data dramatically modifying the way scientific research is carried out. The focus is shifting from the study of ”isolated realities” to the understanding of whole biological systems and the interactions between the huge number of their individual components. From the beginning of this revolution, machine learning immediately appeared as a natural tool for sorting, analyzing, and extracting useful information from these large amounts of data. Unfortunately, some peculiar characteristics of biological data, such as the large number of variables and the low number of samples, challenged even the most robust machine learning algorithms, especially when considering their use in a real clinical setup. This paper shows how the use of simple decision rules can be used to add to state-of-the-art multi-class classifiers a rejection option able to discard samples that do not belong to any of the trained classes. Traditionally, this operation is performed by other rejection methods, like one-class classifiers, which do not perform very well on microarray data. The proposed solution has several advantages:

• it can be easily plugged into an environment widely spread in several research groups;

• it is simple and does not require high computational resources;

• in general it performs better than other available solutions such as those based on one-class classifiers;

• it automatically tunes all parameters for the rejection model, requiring minimum intervention from the user.

## Methods

### Multi-class classifier setup in R

The three considered multi-classifiers (RF, k-NN and N-NET) have been trained in R resorting to the Classification And REgression Training (CARET) package, a set of functions that attempt to streamline the process for creating predictive models in R [30]. There are many different modeling functions in R. Some have different syntax for model training and/or prediction. CARET provides a uniform interface to these functions allowing for standardization of common tasks.

Parameter tuning of each classifier in CARET is done via resampling; every candidate model is evaluated many times using cross-validation. The number of candidate models is set through the setTuneLength parameter. In our experiments we used setTuneLength=5. This value represents a good compromise in terms of computational time of the training phase and accuracy of the results for our experimental setup. Resampling has been performed according to the following parameters that can be set in CARET using the trainControl function:

• method = "LGOCV": evaluation of each candidate model is performed using leave-group-out-cross-validation,

• number = 30: number of resampling for LGOCV,

• p = 0.98: percentage of samples in the training set of each resampling.

Data have been pre-processed before performing classification for normalization and dimensionality reduction.

Near zero variance (NZV) features have been removed from the data-set resorting to the nearZeroVar() function of CARET. The resulting data have been then processed using the preProcess() function of CARET. This function allows us to create an object able to perform centering, scaling and dimensionality reduction using PCA. Normalization is performed based on training data, only.

At this stage the data-set is ready for classification. The classification model for each classifier is built using the train function in CARET, and the class probability estimates for each sample in the test set are computed resorting to the extractProb() function. The way class probabilities are estimated by the extractProb() function is classifier dependent:

• k-NN performs classification based on the k closest training examples. Majority voting is used to predict the target class. The class probability estimate for a class is the number of training neighbors belonging to the class over the k considered neighbors.

• RF is an ensemble classifier that consists of many decision trees. It predicts the class that is the mode of the classes predicted by individual trees. Similarly to k-NN the class probability estimate for a class is the number of individual decision trees predicting the class over the total number of decision trees in the forest.

• N-NET predictions are performed by evaluating the values returned by each of the output neurons (one for each available class). The output layer typically outputs the value of a sigmoid function of the linear combination of hidden layer values representing a posterior probability. This value is used as class probability estimate.

### Threshold setup modules

#### A short overview of the CMA-ES

The covariance matrix adaptation evolution strategy (CMA-ES) is an optimization method first proposed by Hansen, Ostermeier, and Gawelczyk [31] in mid 90s, and further developed in subsequent years [32,33]. It is particularly suited for solving optimization problems where no preliminary hypothesis on the solution can be derived. It is therefore a good choice for our specific problem, in which we want to compute the optimal rejection thresholds with a complete automated approach. In our specific application provided solutions are represented by a vector **x** = (*T_max_*, *T_rej_*, *T_diff_*) containing the three rejection thresholds.

In the CMA-ES, iteration steps are called *generations* due to its biological foundations. The value of a generic algorithm parameter *y* during generation *g* is denoted with *y*^(*g*)^. The mean vector **m**^(*g*)^ ∈ ℝ*^n^* represents the favorite, most-promising solution so far. The *step size σ*^(*g*)^ ∈ ℝ_+_ controls the step length, and the *covariance matrix***C**^(*g*)^ ∈ ℝ^*n*×*n*^ determines the shape of the distribution ellipsoid in the search space. Its goal is, loosely speaking, to fit the search distribution to the contour lines of the objective function *f* to be minimized. **C**^(0)^ = **I**

In each generation *g*, λ new solutions  are generated by sampling a multi-variate normal distribution  with mean 0 (see equation 1).(1)

Where the symbol · ~ · denotes the same distribution on the left and right side.

After the sampling phase, new solutions are evaluated and ranked. **x**_*i*:λ_ denotes the *i^th^* ranked solution point, such that *f*(**x**_1:λ_) ≤ … ≤ *f*(**x**_λ:λ_). The *µ* best among the λ are selected and used for directing the next generation *g* + 1. First, the distribution mean is updated (see equation 2).(2)

In order to optimize its internal parameters, the CMA-ES tracks the so-called *evolution paths*, sequences of successive normalized steps over a number of generations.  is the conjugate evolution path.  is the expectation of the Euclidean norm of a  distributed random vector, used to normalize paths.  is usually denoted as *variance effective selection mass*. Let *c_σ_* < 1 be the learning rate forcumulation for the rank-one update of the covariance matrix; *d_σ_* ≈ 1 be the damping parameter for step size update. Paths are updated according to equations 3 and 4.(3)(4)

 is the evolution path, . Let *c_c_* < 1 be the learning rate for cumulation for the rank-one update of the covariance matrix. Let *µ*_cov_ be parameter for weighting between rank-one and rank-*µ* update, and *c*_cov_ ≤ 1 be learning rate for the covariance matrix update. The covariance matrix **C** is updated (equations 5 and 6).(5)(6)

where OP (**X**) = **XX^T^** = OP(–**X**).

Most noticeably, the CMA-ES requires almost no parameter tuning for its application. The choice of strategy internal parameters is not left to the user, and even λ and *µ* default to acceptable values. Notably, the default population size λ is comparatively small to allow for fast convergence. Restarts with increasing population size have been demonstrated [34] to be useful for improving the global search performance, and it is nowadays an option included in the standard algorithm.

#### Objective functions

Four objective functions have been evaluated in the optimization process, all of them trying to optimize the outcome of the classification process. The optimization process stops when it reaches one of three possible conditions:

1. *f* reaches a predefined lower bound. This represents the best condition corresponding to the identification of the optimum solution;

2. The value of *f* for the current population does not change more than a given value *δ*. The CMA-ES reached a local optimum that cannot be further improved with the current population;

3. The value of *f* for the last *p* populations does not change more than a given value *α* <*δ*. Again the CMA-ES reached a local optimum. In this case despite the solution can be still slightly improved, globally, the increment is not significant and therefore it is not worth continuing the optimization.

Sensitivity and specificity are common indicators of the efficiency of a binary classification test that can be exploited in the definition of the objective function. They are here computed taking into account that the outcome of the classification rule may also produce uncertain results:(7)(8)

Based on these two indicators we tested three objective functions defined as follows:(9)(10)(11)

As required by the CMA-ES that is designed to minimize the objective function, greater values of sensitivity and specificity decrease the value of the objective function. The first function considers the contribution of sensitivity and specificity separately, thus allowing for solutions where mostly only one of the two indicators is maximized. The second and the third functions try to leverage this problem by forcing the optimization towards results where both sensitivity and specificity are equally maximized. In particular *SS*1 seems to be the best objective function able to take into account the relationship between sensitivity and specificity

Similarly to sensitivity and specificity, the F-Score*_β_* is another statistical indicator of the outcome of a binary test considering the precision *p* and the recall *r* of the test. Again when computing *p* and *r* uncertain results should be considered as follows:(12)(13)

The F-Score*_β_* has been exploited as objective function of the CMA-ES as follows:(14)

*FS*1 and *FS*0.5 come from the *FS_β_* where *β* is set to 1 and 0.5 respectively. Experimental results demonstrated that this function is quite inefficient since it tends to privilege increments of TP penalizing TN.

## List of abbreviations used

ALL: acute lymphoblastic leukemia; AML2: acute myeloid leukemia 2; CARET: classification and regression training; CBF-AML: core binding factor acute myeloid leukemia; cDNA: complementary DNA; CI: confidence intervals; CMA-ES: covariance matrix adaptation evolutionary strategy; CLLww: B-cell chronic lymphocytic leukemia wait&watch; CLL: B-cell chronic lymphocytic leukemia; DNA: deoxyriboNucleic acid; DLBCL: diffuse large B-cell lymphoma; FL: follicular lymphoma; FP: false positives; FN: false negatives; k-Means-OC: k-Means one-class classifier k-NN, k-nearest neighbors; k-NN-OC: k-NN one-class classifier LOC, level of confidence; LGOCV: leave group out cross validation MOCV, multi one-class with voting; N-NET: neural network; NZV: near zero variance; Parzen-OC: Parzen one-class classifier PCA, principal component analysis; PCA-OC: PCA one-class classifier ROC, receiver operating characteristic; RF: random forests; Sens: sensitivity SOCMT, single one-class with multiple targets; Spec: specificity SVDD, support vector data description; SVM: support vector machine; SVM-OC: SVM one-class classifier TP, true positives; TN: true negatives.

## Competing interests

The authors declare that they have no competing interests.

## Authors contributions

AB coordinated the overall design and implementation of the method, and performed the experimental results analysis. SD participated in the design of the decision rules, in the definition of the evolutionary method for the threshold computation and defined the experimental validation. GP conceived and implemented the proposed decision rules and carried out the classification experiments. AS defined and implemented the evolutionary method for the computation of the rejection thresholds, and performed the related experiments for its application on the selected case study. HH performed the statistical analysis for the validation of the results. All authors contributed to draft the manuscript. All authors read and approved the final manuscript.
